# 

**Published:** 1847-04

**Authors:** 


### NOTICE TO READERS AND CORRESPONDENTS.

Original communications from Drs. Mead and Fitch have been received and will appear in our next.

We have received New Elements of Operative Surgery; ALF. A. L. M. VELPEAU Prof., etc. Carefully revised, augmented, etc., by VALENTINE MOTT, Prof., etc. First American from last Paris edition. Translated by P. S. TOWNSHEND, M. D. New York: Sam'l. S. and Wm. Wood. 1847. pp. 1162 octavo, with an atlas containing 22 plates quarto. (From the Publishers.)

Report of the Pennsylvania Hospital for the Insane, for the year 1846; by THOMAS S. KIRKBRIDE, M. D., Physician to the institution. 8vo. pp. 36

Fourth Annual Report of the Managers of the State Lunatic Asylum, made to the Legislature of New York, February 2d, 1847. 8vo. pp. 80

Twenty-sixth Annual Report of the Bloomingdale Asylum for the Insane; by PLINY EARLE, M. D., Physician to the Asylum. 1847. 8vo. pp. 32.

Catalogue of Books on Medicine, etc., etc., for sale by Samuel S. and William Wood, No. 261 Pearl street, New York.

We have received in exchange the following periodicals:

New York Journal of Medicine, etc., New York.
The Annalist, a Record of Practical Medicine, New York.
New York Medical and Surgical Reporter, New York.
The Medical Examiner, etc., Philadelphia.
The Medical News end Library, Philadelphia.
The Western Journal of Medicine and Surgery, Louisville, Ky.
The Western Lancet and Medical Library, Lexington, Ky.
The Southern Medical and Surgical Journal, Augusta, Georgia.
The Boston Medical and Surgical Journal, Boston, Mass.
The Missouri Medical and Surgical Journal, St. Louis, Missouri.
The St. Louis Medical and Surgical Journal, St. Louis, Missouri.
The Practical Educator and Journal of Health, Boston.
Stockton and Co.'s Dental Intelligencer, Philadelphia.
La Lancette Canadienne, Montreal, L. C.

Also, Announcement of the Medical Institute of Philadelphia, for 1847, with a Catalogue of Students.

Catalogue of Jefferson Medical College of Philadelphia, Session of 1846–'47.

Announcement and Catalogue of the University of Louisville, for 1847.

---

### CONTRIBUTORS TO THE ILL. AND IND. MED. AND SURG. JOUR.

S. G. Armor, M. D., Rockford, Ill.
A. H. Howland, M. D., Ottawa, Ill.
A. G. Henry, M. D., Pekin, Ill.
Daniel Stahl, M. D., Quincy, Ill.
Edward Mead, M. D., Geneva, Ill.
H. S. Huber, M. D., Chicago, Ill.
David Prince, M. D., Jacksonville, Ill.
J. F. Henry, M. D., Burlington, Iowa.
Jno. McLean, M. D., Jackson, Mich.
Edward Lewis, M. D., Jackson, Mich.
Ira C. Bachus, M. D., Jackson, Mich.
J. G. Conwell, M.D., Spring Arbor, Mich.
S. B. Thayer, M. D., Battle Creek, Mich.
E. Deming, M. D., Lafayette, Ind.
G. N. Fitch, M. D., Logansport, Ind.
Elias Fisher, M. D. Waynesville, Ohio.

### PROSPECTUS

OF THE

ILLINOIS AND INDIANA

### MEDICAL AND SURGICAL JOURNAL.

The second volume, (new series,) of this work will be issued regularly on the first of every other month, beginning with May, simultaneously at Chicago and Indianapolis. It will be neatly printed, with new type, on good paper; each number containing about 96 papes octavo; and will at the end of the year form a volume of about 575 pages, with title-page and index complete.

The more liberal patronage of the Journal encourages us to renewed exertions, while the addition of a number of distinguished physicians to our list of correspondents, will enable us to furnish a greater variety under the head of original communications.

The diseases peculiar to the West and N. West shall claim our especial consideration, which will render it more particularly valuable to the physicians of this region.

Contributions to our pages by physicians, are earnestly solicited.

Terms—Two dollars per annum, *in advance*, which may be paid to either of the editors, or our authorized agents.

Remittances free or post-paid may be made at our risk.

A few copies of the last volume can be furnished if desired.

Agents—Mr. Sample Loftin, of Indiana; Mr. Henry Donaldson for Bureau and surrounding counties, Illinois.

